# Intestinal Donation and Utilization: Single-Center Analysis Within Eurotransplant

**DOI:** 10.3389/ti.2023.11371

**Published:** 2023-08-21

**Authors:** Mathias Clarysse, Tim Vanuytsel, Emilio Canovai, Diethard Monbaliu, Laurens J. Ceulemans, Jacques Pirenne

**Affiliations:** ^1^ Department of Abdominal Transplant Surgery and Coordination, University Hospitals Leuven, Leuven, Belgium; ^2^ Abdominal Transplant Surgery, Department of Microbiology, Immunology and Transplantation, KU Leuven, Leuven, Belgium; ^3^ Leuven Intestinal Failure and Transplantation Center, University Hospitals Leuven, Leuven, Belgium; ^4^ Department of Gastroenterology and Hepatology, University Hospitals Leuven, Leuven, Belgium; ^5^ Translational Research Center for Gastrointestinal Disorders, Doctoral School of Biomedical Sciences, KU Leuven, Leuven, Belgium; ^6^ Department of Thoracic Surgery, University Hospitals Leuven, Leuven, Belgium; ^7^ Laboratory for Respiratory Diseases and Thoracic Surgery, Department of Chronic Diseases, Metabolism and Ageing, Faculty of Medicine, KU Leuven, Leuven, Belgium

**Keywords:** awareness, intestinal transplantation, organ donation, organ allocation, utilization

## Abstract

Intestinal donor criteria are classically kept strict, thereby limiting donor supply. Indications for intestinal transplantation (ITx) are rare, but improved outcome and new emerging indications lead to increased demand and relaxing donor criteria should be considered. We sought to compare the donor criteria of intestines transplanted at our center with predefined (per protocol) criteria, and to determine how relaxing donor criteria could impact the potential donor pool. Donor criteria used in 22 consecutive ITx at our center between 2000 and 2020 were compared with predefined criteria. Next, multiorgan donors effectively offered by our Donor Network to Eurotransplant between 2014 and 2020 were retrospectively screened, according to predefined and effectively used intestinal donation criteria. Finally, utilization rate of offered intestines was calculated. In our ITx series, the effectively used donor criteria were less strict than those initially predefined. With these relaxed criteria, a favorable 5-year graft/patient survival of 75% and 95%, respectively was reached. Applying these relaxed criteria would lead to a 127% increase in intestinal offers. Paradoxically, 70% of offered intestines were not used. In conclusion, a significant increase in intestinal donation could be obtained by relaxing donor criteria, while still achieving excellent outcome. Offered intestines are underutilized.

## Graphical Abstract

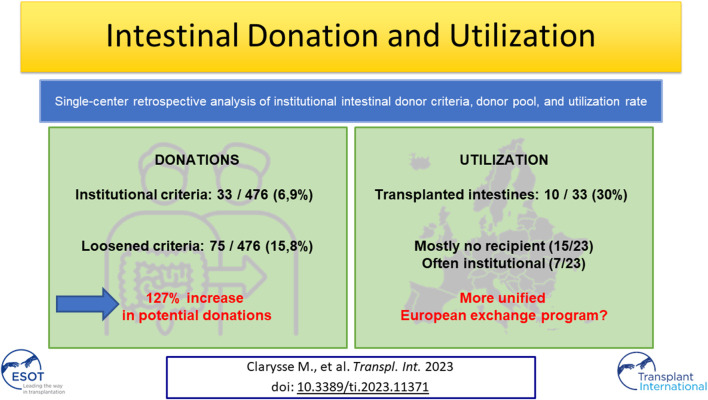

## Introduction

Intestinal transplantation (ITx) is indicated in patients with intestinal failure and life-threatening complications of parenteral nutrition [1, 2]. So far, ITx has only been advocated as a “salvage procedure,” due to the complexity of the procedure and to outcomes traditionally inferior to other solid organ transplants. However, patient survival reaching 90% and 75% at 1- and 10-year post-transplantation has been reported [2–5]. In addition, new indications like extensive mesenteric thrombosis or other diffuse abdominal diseases, necessitating multivisceral transplantation, are emerging [6]. As a consequence, the demand for suitable donor intestines is increasing.

The intestine is very susceptible to ischemia and preservation injury and for this reason, ITx centers usually only use so called “excellent donors” e.g., donors with very strict predefined criteria. These donors are rare and waiting time can be long. Within United Network for Organ Sharing (UNOS), more than 50% of the listed patients wait more than 1 year prior to transplantation and the risk of deterioration and mortality on the list is high [4]. Since the start of an ITx waiting list in Eurotransplant (ET) on 1st October 2012, a mortality rate of 27% (*n* = 30/113) has been observed, and 3% of the patients (*n* = 3/113) were delisted because deemed unfit for ITx (personal communication with ET). “Extended” donor criteria are now accepted for the majority of solid organs [donation after circulatory death (DCD), advanced age, prolonged intensive care unit (ICU) stay, co-morbidity …] [7]. Because the intestine is extremely vulnerable to warm ischemia, DCD donors are not routinely used and donation after brain death (DBD) donors represent the largest source of intestinal grafts. In several European countries, there is a shift from DBD towards DCD donors, thereby further reducing the availability of intestinal grafts. For the aforementioned reasons, relaxing the criteria for intestinal donation is becoming necessary.

At the start of our program, we predefined strict donor criteria and we now wanted to determine whether these criteria had actually been respected in our ITx series. Secondly, we determined, in our own donor pool, how slightly relaxing donor criteria would increase the number of intestinal grafts. Lastly, we studied the utilization rate of offered intestines.

## Patients and Methods

### UZ Leuven Donor Network

Belgium has opt-out legislation for organ donation since 1986 and no separate informed consent is needed for intestinal donation. Each of the seven Belgian transplant centers has its own procurement organization, consisting of the respective transplant university hospitals and their own network of cooperating “donor” hospitals. The UZ Leuven Donor Network for organ procurement includes The University Hospitals Leuven and its 37 cooperating hospitals across Flanders, Belgium (LSGO). Belgium is part of ET, and solid organs procured in the LSGO are allocated by ET. Allocation of intestinal grafts to ET waitlisted patients occurs in a patient-driven manner, over three active ITx centers in three countries in 2022. In the time period of the study, 2014–2020, there were seven active ITx centers in four countries.

### Predefined Intestinal Organ Donor Criteria at Our Center

At the start of our ITx program in 2000, intestinal organ donor criteria were predefined and are summarized in Table 1 [8]. All deceased intestinal donors should be DBD, age < 50 years, weight ≤ 80 kg, Body Mass Index (BMI) ≤ 25 kg/m^2^. Blood group matching is identical or compatible. Exclusion criteria included smoking, alcohol, drug abuse, and diabetes. Liver and kidney function tests must be normal. Recent cardiac arrest, cardiopulmonary resuscitation (CPR), or hypotensive episodes are excluded. The donor should be hemodynamically stable, with minimal transfusions and inotropic support (<2 drugs at low dosage). ICU stay should be less than 5 days. As a result, intestinal donors are often multi-organ donors (Heart, Lungs, Liver, Pancreas, Kidneys).

**TABLE 1:** Comparison between predefined and effective intestinal donation criteria in Leuven.

			Demographics and medical history										Lab results									
			Type of donor	Age (years)	Weight (kg)	BMI (kg/m^2^)	Weight ratio	Blood group (ABO) compatibility	Smoking	Alcohol	Drug	Diabetes	Highest AST (U/L)	Highest ALT (U/L)	Highest bilirubin (mg/dL)	Highest INR	Highest amylase (U/L)	Highest lipase (U/L)	Highest creatinine (mg/dL)	Last serum [Na+]		
Predefined	Leuven		DBD	<50	≤80	≤25		Compatible	No	No	No	No	Acceptable for transplantation							<155		
Effective	Leuven	2000–2020	DBD	16 (1–37)	50 (12–75)	19.6 (11–26)	0.9 (0.5–1.5)	Compatible	Yes	Yes	No	No	63 (12–269)	25 (9–198)	0.4 (0.05–3.4)	1.27 (0.9–1.7)	105 (16–603)	16 (6–101)	0.68 (0.2–1.3)	148 (130–157)		
N° of patients exceeding predefined criteria			0	0	0	1	0	0	2	1	0	0	0	0	0	0	0	0	0	1		

			Hemodynamics						Inotropics and transfusion and ICU stay									Multi-organ donor				
			CPR	CPR time (min)	Cardiac arrest	Arrest time (min)	Hypotens episodes	Hypotens time (min)	NOR	γ of NOR	DOB	γ of DOB	EPI	γ of EPI	Packed cells	# of packed cells	ICU stay (days)	Heart offered	Lungs offered	Liver offered	Pancreas offered	Kidneys offered
Predefined	Leuven		No	0	No	0	No	0	Yes	<0.1	Yes	<5					<5	Yes	Yes	Yes	Yes	Yes
Effective	Leuven	2000–2020	Yes	0 (0–30)	Yes	0 (0–45)	Yes	0 (0–15)	Yes *n* = 15	0.1 (0.01–0.68)	Yes *n* = 2	2 (1–3)	Yes *n* = 1	0.14	Yes *n* = 8	0 (0–5)	2 (1–9)	Yes *n* = 20	Yes *n* = 20	Yes *n* = 22	Yes *n* = 22	Yes *n* = 22
N° of patients exceeding predefined criteria			3	3	3	3	2	2	0	8	0	0	0	0	0	0	2	2	2	0	0	0

Green = equal; red = predefined violation. ALT, alanine transaminase; AST, aspartate transaminase; BMI, body mass index; DBD, donation after brain death; DOB, dobutamine; EPI, epinephrine; ICU, intensive care unit; INR, international normalized ratio; [Na+], Serum Sodium Concentration; N°, number; NOR, norepinephrine; REA, resuscitation; γ, µg/kg/min.

### Donor Criteria of Intestines Transplanted at Our Center

A retrospective analysis of our prospectively collected ITx database (October 2000—September 2020) was performed. One living donor ITx was excluded and only deceased ITx recipients (*n* = 22) were analyzed. Data analyzed were: donor type, age, weight, length, BMI, donor/recipient weight ratio, ABO blood group compatibility, smoking/alcohol/drug abuse, diabetes, latest lab results (Aspartate Transaminase (AST), Alanine Transaminase (ALT), total bilirubin, International Normalized Ratio (INR), amylase, lipase, creatinine, [Na+], CPR time, cardiac arrest time, hypotensive episodes, inotropic use and dosage, number of transfused packed cells, ICU stay, and other organs offered (heart, lungs, liver, pancreas, kidneys).

### Retrospective Screening of Donor Pool According to Predefined Versus Actually Used Donor Criteria

Data of donors offered by LSGO to ET during a 6-year period (1st January 2014—31st December 2019), and prospectively collected in an *ad hoc* donor database, were analyzed. Data included: donor type, age, weight, length, BMI, ABO and rhesus blood group, smoking/alcohol/drug abuse, diabetes, virology status (human immunodeficiency virus, hepatitis B, hepatitis C), CPR time, cardiac arrest time, hypotensive episodes, inotropic use (number and dosage), transfused packed cells, ICU stay, organs offered and transplanted (heart, lungs, liver, pancreas, kidneys, and intestine), and reasons for not offering or for refusing the intestine.

This donor cohort was screened, first according to the aforementioned predefined intestinal donor criteria, and second according to the criteria effectively applied in our ITx program and defined in the first part of the study.

#### Statistics

Data were collected using Excel (Microsoft Office 2019). Results are reported as median (range). Subgroup analysis was performed by non-parametric Mann-Whitney Test in GraphPad Prism version 9.0.0 for Windows (GraphPad Software, La Jolla, CA, United States). *p*-value < 0.05 was considered statistically significant.

#### Ethics

All ITx patients gave consent for database recording of transplant-related data and their use for research purposes. This study was approved by the ethics committee of University Hospitals Leuven, Belgium (S63306) and was conducted according to the revised version of the Declaration of Helsinki (October 2013, Brazil).

## Results

### Leuven ITx Series

**FIGURE 1:**
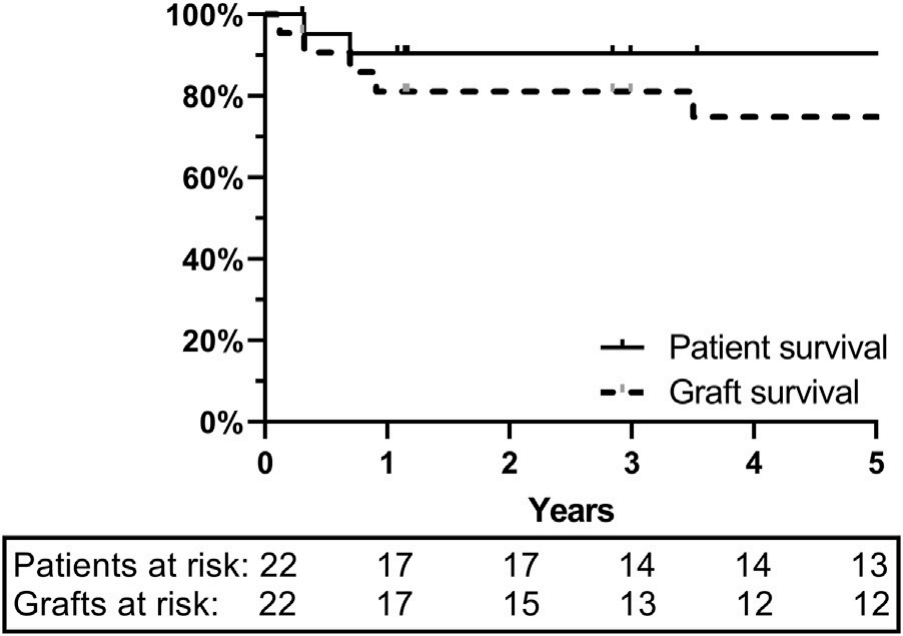
One- and 5-year graft and patient survival at University Hospitals Leuven: ITx cohort of 22 patients.

Between 19 October 2000 and 01 September 2020, 22 deceased ITx were performed. Ten were isolated ITx, five multivisceral transplants, and seven combined liver-ITx. Two were retransplants. Eight donors (36%) were from our local network (LSGO) and the remaining 14 (64%) from ET. Pre-, peri-, and post-transplant surgical and medical management have been described extensively elsewhere [8]. One- and 5-year graft and patient survival were 81%/75% and 95%/95%, respectively (Figure 1).

### Intestinal Donor Criteria Used in Our ITx Cohort are Less Strict Compared to Predefined Criteria

All donors used for ITx were DBD with a median age of 16 years (1–37). Median weight was 50 kg (12–75). BMI was 19.6 kg/m^2^ (11–26). BMI was higher than the predefined maximum of 25 kg/m^2^ in one donor. Donor/recipient weight ratio was 0.9 (0.5–1.5). Blood group was compatible in 5 and identical in 17. Smoking and alcohol abuse were present in 2 and 1 donors, respectively. Drug abuse and diabetes were not reported. The latest lab results were acceptable for liver transplantation and kidney transplantation, and last [Na+] was 148 mmol/L (130–157). The predefined maximum [Na+] was overruled in one donor with a [Na+] of 157 mmol/L. In three donors, CPR had been performed for 20 min (10–30) and cardiac arrest time in these donors was 25 min (5–45). Hypotensive episodes of 10 min were noted in two donors (5–15). Norepinephrine was used at a dosage of 0.1 μg/kg/min (γ) (0.0155–0.68) as single inotropic in 13 donors and in combination in 2 others. In eight donors, norepinephrine dosage exceeded the predefined maximum of 0.1γ. Dobutamine was used in two donors, in one as single inotropic, at a dosage of 2γ (1–3). In one donor, epinephrine was used, as second inotropic, at a dosage of 0.14γ. Packed cells transfusion was given in eight donors with a median of 2 units of packed cells (1–5). Median ICU stay was 2 days (1–9) and exceeded the predefined maximum in two donors, with 5 and 9 days, respectively. All abdominal organs were offered in all donors. Heart and lungs were not offered in three donors due to thoracic trauma, atrial fibrillation, and for unknown reason (Table 1).

In 55% (*n* = 12/22) of these effective intestinal donors, the strict predefined criteria had been overruled for BMI, CPR, inotropic use, and ICU stay.

### DBD Pool Filtered by Predefined ITx Criteria

From 1st January 2014, to 31st December 2019, the LSGO had referred 664 donors to ET. Of them, 188 (28%) were DCD and 476 (72%) were DBD donors.

**FIGURE 2:**
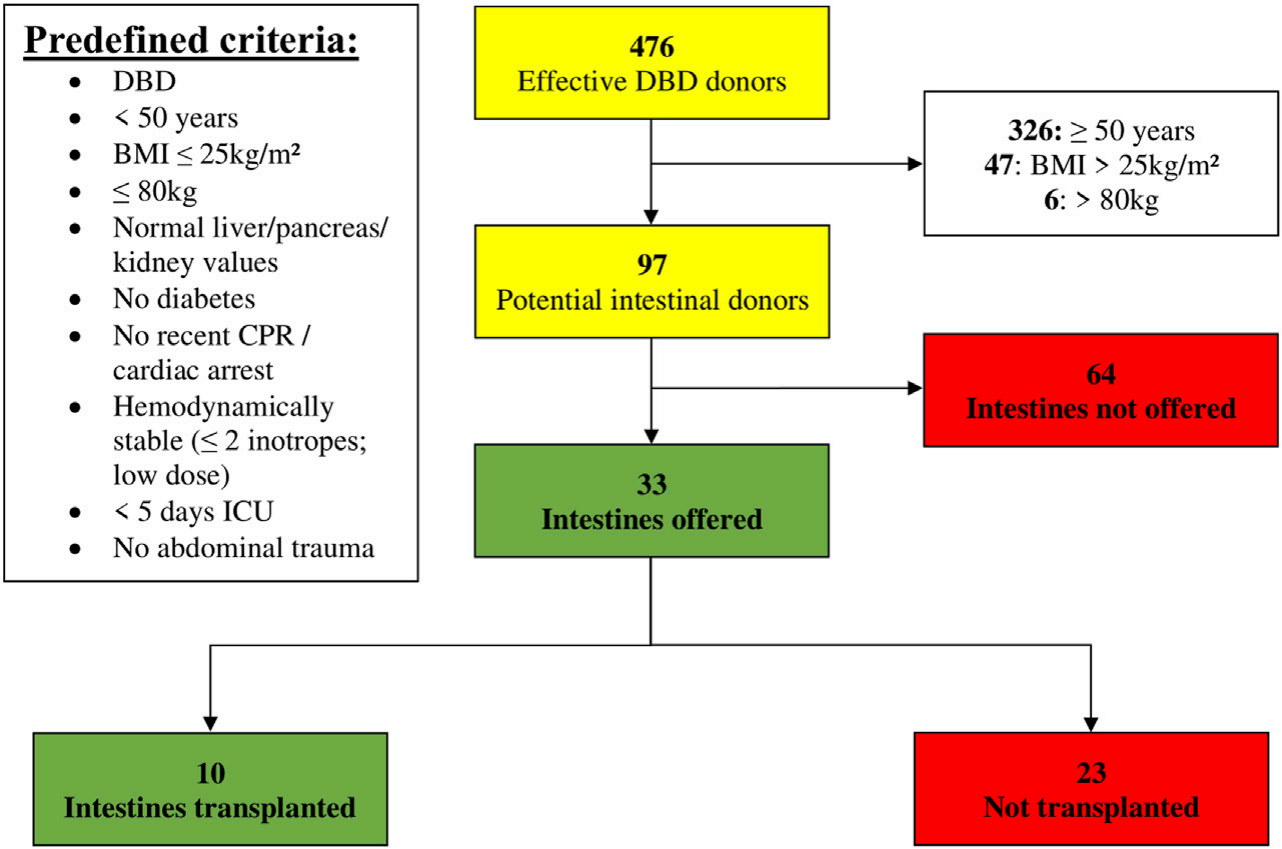
Donor pool (01 January 2014–31 December 2019) filtered by predefined criteria (BMI, Body Mass Index; CPR, Cardiopulmonary Resuscitation; DBD, Donation after Brain Death; ICU, Intensive Care Unit).

Of the 476 DBD donors, 68% (*n* = 326/476) were excluded for age ≥ 50 years (Figure 2). Of the remaining 150 donors, 47 were excluded due to a BMI > 25 kg/m^2^. Six additional donors were excluded for weight > 80 kg. Hence, one out of five DBD donors (*n* = 97/476; 20%) matched the predefined theoretical anthropomorphic criteria for ITx. Thirty-three intestines from these donors (*n* = 33/97; 34%) were offered to ET. Ten were effectively transplanted within ET, of which 6 in our own center. Out of the 33 offered intestines, only 10 were transplanted and 23 could not be allocated, representing a utilization rate of 30.3% (*n* = 10/33).

In 64 out of 97 donors (66%), the intestine was not offered, for which the reasons are listed in Table 2. Of these 64 donors, predefined criteria were not met in 49 (77%). The three main reasons were > 10 min of CPR (*n* = 21/49; 43%), ICU stay of > 5 days (*n* = 8/49; 16%) and high inotropic need (*n* = 6/49; 12%). In the 15 remaining donors, the most important reasons for not offering the intestine were low cardiac ejection fraction on ultrasound in 3 (5%) and malignancy in 2 (3%) (a grade IV glioblastoma in one and a grade I–II astrocytoma with previous tumor surgery, in another).

**TABLE 2:** Reasons for not offering the intestine.

64 intestines not offered
Predefined criteria (*N* = 49)
×21 > 10 min CPR
×8 > 5 days ICU
×6 high inotropic need
×5 > 20 min hypotension
×4 drug abuse
2 × 4 units packed cells
×1 abdominal trauma
×1 diabetes mellitus type 1
×1 diabetes mellitus type 2
Other reasons (*N* = 15)
×3 low cardiac ejection fraction
×2 malignancy
×1 extreme height + weight
×1 gastric bypass surgery
×1 hemochromatosis
×1 infectious
×1 legal issues
×5 unknown

CPR, cardiopulmonary resuscitation; ICU, intensive care unit; N, Number.

In 55 of these non-offered donors (86%), only one exclusion criterium for not offering the intestine (CPR) was present. In 7 (11%), two criteria (among them CPR time, hypotension, ICU stay, and/or inotropic need) were present. In 2 (3%), 3 exclusion criteria were present (CPR time, hypotension, and inotropic need).

In 33 donors, the intestine was offered despite the presence of one exclusion criteria in 20 (61%) and two exclusion criteria in 4 (12%). Overruled criteria were mainly: CPR time, hypotension, ICU stay, and/or inotropic need. Other reasons for overruling criteria were noted in four donors but were not specific to intestinal donation: bacterial meningitis in two, meningioma in one, and a thyroid tumor in another (Supplementary Table S1).

In 5 out of the 10 transplanted intestines (50%), one predefined criterium was overruled (CPR time, ICU stay, or inotropic need) and in one donor (10%) two criteria were overruled (prolonged ICU stay and high inotropic need) (Supplementary Table S2).

### Reasons for Not Using Offered Intestines

Of the 23 refused donor intestines, 7 (30.4%) had directly been offered to our own center but could not be used for organizational reasons. They were then offered to ET but could not be transplanted either. Fifteen intestines directly offered to ET (65%) could not be used, potentially due to absence of suitable recipients within ET or for organizational reasons (Table 3). Finally, in one donor, a low cardiac ejection fraction was diagnosed after the intestine was offered and subsequently declined for transplantation.

**TABLE 3:** Reasons for not transplanting the offered intestine.

23 intestines offered—not transplanted
×15 no recipients
×7 capacity reasons in Leuven
×1 low cardiac ejection fraction

To determine whether donor factors would account for the non-acceptance of intestinal offers, we compared the donor data of transplanted vs*.* not transplanted intestines (Table 4). No difference was seen in anthropomorphic criteria (age, BMI, weight, height) and in ICU stay. In the non-transplanted cohort, there were two children under 1 year of age, with a very low BMI and weight. Other reasons are reported in four donors, as mentioned above (two bacterial meningitis, one meningioma and one thyroid tumor).

**TABLE 4:** Donor criteria in transplanted versus “no-recipients” cohort.

	Transplanted cohort (*N* = 10)				No-recipients cohort (*N* = 22)				*p*-value
Age (years)	21 (1–41)				26 (0–46)				0.6964
BMI (kg/m^2^)	20.7 (16.6–23.1)				21.4 (10.7–24.8)				0.7410
Weight (kg)	67.5 (12–75)				62.5 (3–80)				0.5003
Height (cm)	180 (85–185)				171 (53–185)				0.1497
ICU stay (days)	2 (0–6)				2 (0–9)				0.6546
Blood group	O	A	B	AB	O	A	B	AB	
	4	6	0	0	10	9	2	1	

Numbers represented as median (range). *p*-value < 0.05 was considered statistically significant. CPR, cardiopulmonary resuscitation; ICU, intensive care unit; N, Number.

### Donor Pool Filtered by Effectively Applied Criteria and Potential Impact on Intestine Donor Pool

The predefined criteria were extended with the following effectively applied criteria: BMI ≤ 26 kg/m^2^, hemodynamic parameters (CPR < 45 min; hypotensive episodes, norepinephrine < 0.68γ, transfused packed cells), and ICU < 10 days.

**FIGURE 3:**
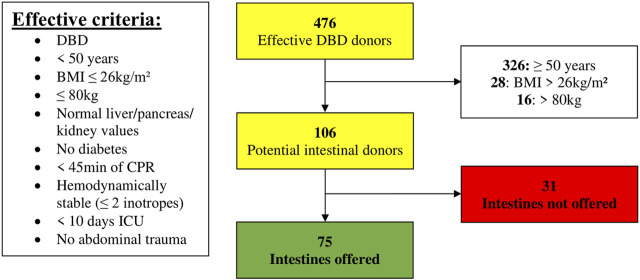
Donor pool (01 January 2014–31 December 2019) filtered according to effective criteria (BMI, Body Mass Index; CPR, Cardiopulmonary Resuscitation; DBD, Donation after Brain Death; ICU, Intensive Care Unit).

Of the 476 DBD donors, 68% (*n* = 326/476) were excluded for age ≥ 50 years. Of the remaining 150 donors, 28 were excluded due to a BMI > 26 kg/m^2^ and an additional 16 donors were excluded for weight > 80 kg. Hence, 22% of DBD donors (*n* = 106/476) met the effective anthropomorphic criteria. Seventy-five donors (71%) met all of the effectively applied criteria and could have been potential intestinal donors (Figure 3).

Only 31 non-offered donors would then remain. Twenty-three percent had a CPR ≥ 45 min (*n* = 7/31) and another 23% had an ICU stay ≥ 10 days (*n* = 7/31). Drug abuse would still lead to the exclusion of 16% (*n* = 5/31) and low cardiac ejection fraction to 10% (*n* = 3/31). Other reasons are mentioned in Table 5. In all these 31 non-offered donors, there was only one reason why the intestine would not have been offered.

**TABLE 5:** Reasons for not offering the intestine according to effective criteria analysis.

31 intestines not offered
Effective criteria (*N* = 22)
×7 ≥ 45 min CPR
×7 ≥ 10 days ICU
×5 drug abuse
×1 abdominal trauma
×1 diabetes mellitus type 1
×1 diabetes mellitus type 2
Other reasons (*N* = 9)
×3 low cardiac ejection fraction
×2 gastric bypass surgery
×2 infectious
×1 hemochromatosis
×1 legal issues

CPR, cardiopulmonary resuscitation; ICU, intensive care unit; N, Number.

If the effective criteria would have been applied, this would have resulted in an additional 42 intestinal offers. Main reasons for additional inclusion would have been CPR 10–45 min in 17 donors, 5 donors with hypotensive episodes, and 7 with inotropic need (Table 6). Inclusion of these 42 additional intestinal offers, would result in a potential donor increase of 127%. In 98% (41/42) of these additional donors, application of the effective criteria would have resulted in only one violation to the predefined criteria. In the latter one, there would have been two violations: CPR time exceeding 10 min (i.e., 15 min) and usage of two inotropics (0.4γ norepinephrine and 0.3γ epinephrine).

**TABLE 6:** Additional intestinal donor offers when using effective criteria.

42 potential extra offers
Effective criteria (*N* = 33)
17 × 10–45 min CPR
×7 high inotropic need
×5 hypotension
2 × 4 units packed cells
2 × 5–10 days ICU
Other reasons (*N* = 9)
×5 unknown
×3 malignancy
×1 extreme height + weight

CPR, cardiopulmonary resuscitation; ICU, intensive care unit; N, Number.

## Discussion

The only limiting factor for wider application of solid organ transplantation is the shortage of suitable organs. To meet the higher demand, donor criteria have been progressively relaxed over time. The intestine stands in stark contrast because intestinal donor criteria have usually remained very strict, mostly because the intestine is highly vulnerable to ischemia. Here, we show that outcomes similar to other solid organ transplants can be obtained despite using slightly relaxed criteria. By applying these relaxed criteria, we could substantially increase the pool of intestinal donors. Importantly, we found that a substantial portion of offered intestines are not utilized, suggesting the need for a European-wide intestinal donor organ sharing program.

At first sight, the need for extension of the intestinal donor pool appears less urgent than for other solid organs. Indeed, ITx remains a rare procedure representing less than 0.5% of the overall solid organ transplant activity. That is because the incidence of intestinal failure is much lower compared to failure of other organs and, in case of “uncomplicated” intestinal failure, parenteral nutrition is still the first treatment option [9]. Obstacles to wider application of ITx are the complex surgical and immunobiological challenge that the transplantation of this naturally infected and immunologically active organ represents, and the reported results which were historically inferior -on average-to other solid organ transplants. This has contributed in some reluctance to refer patients for ITx [10]. However, results similar to other solid organ transplants can now be achieved in experienced centers [4]. Excellent outcome, improved quality of life and the proven cost-effectiveness of ITx versus parenteral nutrition (similar to kidney Tx vs*.* dialysis) is an incentive to propose ITx earlier in the course of intestinal failure [11]. Finally, new indications for intestinal and multivisceral transplantation are emerging such as splanchnic thrombosis and certain tumors [6]. For all these reasons, the demand of suitable intestinal grafts is increasing.

Among solid organs, criteria for intestinal donation are the strictest. This is due to the extreme vulnerability of the bowel to ischemia and reperfusion injury and the wish “not to add additional risks to an already high-risk” procedure. According to the majority of published criteria, age limits are set around 50 years, weight at 80 kg, and BMI at 25–28 kg/m^2^. Liver and kidney function are to be normal, and only limited resuscitation time is accepted. An ICU stay of less than a week and cold ischemia time of max 9 h are other standard cut offs (Supplementary Table S3) [8, 12–16]. At our center, we defined strict criteria for intestinal donation at the onset of our program (Table 1, Supplementary Table S3) [8].

By retrospectively analyzing the characteristics of the intestinal grafts we actually procured and transplanted, we observed that we had overruled our own center-predefined strict criteria in 55%. The most frequently overruled criteria were BMI, CPR, inotropic dosage, and ICU stay. Of note, overruling was more frequent in more recent years which suggests a learning and experience effect (data not shown). When comparing the outcome of our “strict” vs*.* “relaxed” donors, no difference was observed (data not shown). Our overall 5-year patient survival of 95% compares favorably to the international registry, suggesting that having slightly relaxed the donor criteria had no impact on outcome in our program [17].

Historically, hemodynamically unstable donors were deemed unfit for intestinal donation, as the intestine is extremely sensitive to ischemia [18–20]. However, donors with an episode of cardiac arrest and CPR time of up to 52min have been used successfully in different centers [16, 21, 22]. In our series, we used intestines from donors with a median CPR time of 20 min and maximum up to 45 min. Therefore, pre-procurement cardiac arrest and CPR should not necessarily exclude intestinal donation. In addition to CPR, high inotropic need is another reason for excluding intestinal donation. However, data from UNOS suggest that donor intestines exposed to prolonged periods of hypotension were not necessarily predestined to inferior outcome [22]. And with adequate management, inotropics can be weaned or reduced before procurement. In our program, we accepted donors with short hypotensive episodes, limited amount of packed cells transfusion, and donors on no more than two inotropics in acceptable dosages.

Another discriminatory factor in our predefined criteria is ICU stay < 5 days. In literature, mostly 1 week is used as upper limit [16]. However, longer ICU stays have been reported without a clear impact on outcome [21, 22]. Hence, a prolonged stay on ICU should not *per se* limit intestinal organ donation if other criteria are acceptable.

Another option to increase the intestinal donor pool is to accept older donors. The arbitrary upper age limit has usually been fixed at 50 years [13, 14, 16, 23]. However, several publications report successful ITx with donors older than 50 years [13, 14, 24]. Accordingly, age criteria for intestinal donation have been increased to 60 years in Japan and to 65 years in the United Kingdom [22, 25].

Based on our findings and the data published by others, we recommend a slight extension of the intestinal donation criteria. Donor age up to 60 years, BMI up to 28 kg/m^2^ (if donor/recipient size mismatch allows it), ICU stay up to 10 days, limited inotropic usage, previous episodes of hypotension, short period of cardiac arrest and CPR, and limited packed cells transfusions should -separately- not be seen as absolute contraindication for intestinal donation, particularly for patients already more than 1 year on the waiting list or who need a suitable organ more urgently [22].

We showed that the pool of suitable bowels could be substantially enlarged by using these slightly relaxed donor criteria. Indeed, applying these extended criteria to our whole potential donor pool resulted in a 127% increase in intestinal donors*.*

Of note, a multivisceral graft has been recently procured in a DCD donor after normothermic regional reperfusion (NRP) and the transplant was successful [26]. This strategy may allow access to an important pool of currently not utilized intestinal grafts. Especially for extreme young (<1 year of age) recipients, waiting time has more impact on the outcome after ITx. In our cohort, there were no such young recipients and only two extreme young donors (<1 year of age) were offered for transplant. When analyzing the LSGO donor pool of 664 for suitable DCD donors, 11 additional potential intestinal donors could be found with the predefined criteria (Supplementary Figure S1). By applying the effective criteria, 27 potential intestinal DCD donors could be withheld (Supplementary Figure S2). However, more experience on DCD-NRP for ITx is obviously needed.

Strikingly, we noticed that 70% of the intestines offered by our network, were ultimately not used for transplantation. These organs did not differ with regard to anthropometric data and ICU stay, from those grafts that were effectively used. We see two reasons for this underutilization. First, a substantial number of intestinal offers were declined for organizational reasons. Indeed, ITx and procurement require full mobilization of experienced transplant surgeons, transplant anesthesiologists, and gastroenterologists and these are not necessarily permanently standby. On the other, several heart and lung procurement/transplant teams fear inferior outcomes of their graft, if an intestinal graft is concomitantly procured and if special donor preprocurement therapies were initiated. However, the study by Farinelli et al. showed that these intestinal donor preprocurement therapies might even be beneficial for other transplanted organs, without impacting allocation, quality or long-term outcome [27]. ITx may also compete with other organ transplant activities for theater and personnel. In COVID times, travel restrictions for the procurement team and intensive care capacity to take care of these highly demanding patients, further limits the organizational possibilities. Between 1st January 2014, and 31st December 2020, 28 intestinal offers offered by all ET centers were declined explicitly for capacity reasons [28]. In our own center, about 25% of the offered donors had to be declined for organizational reasons as well. The permanent availability of highly specialized ITx services for a small number of patients is challenging and this pleads for more centralization of the procedure.

Second, one intestinal offer at a given time may not necessarily fit all recipients. It is likely that perfectly transplantable intestines were turned down for size-, age-, CMV-mismatch, or other surgical or medical reasons. Size-mismatch is very common in ITx. Most recipients have had previous abdominal surgery and multiple intestinal resections, leaving them with little abdominal domain. Concerning the abdominal domain, there is an important difference whether the transplant graft is liver-containing (combined liver-intestinal or multivisceral) versus isolated small bowel. This limitation can be overcome -to a certain extent-by techniques such as fascia or even full thickness abdominal wall transplantation, graft reduction, etc. [29]. However, these are not commonly used as it further complicates the surgical procedure. It is possible that intestines could not be allocated due to absence of a blood group identical or compatible recipient, and not all centers accept blood group compatible grafts.

Offering these unused intestines outside ET should be considered to increase organ utilization and optimize donor-recipient matching, thereby reducing waiting time and associated mortality. Such cross-program exchange structures already exist for other organs between allocation organizations such as NHSBT (United Kingdom) and Scandiatransplant. Rushton et al. already suggested the possibility to implement a formalized European-wide intestinal donor organ sharing program [25]. A prerequisite for such a European exchange is to keep cold ischemia time short, which would require excellent coordination. Potentially, this could be performed by looking for a second, back up, recipient within or outside ET, at the moment of allocation, in case the intestinal graft gets turned down for own usage by the explant team.

A limitation of our study is that -in all surveyed donors-, a specific reason for not offering the intestine was sought in retrospect. We cannot exclude that in several cases, one simply “forgot” to offer the intestine. That is because ITx is a relatively poorly known activity. We cannot quantify this, but we suspect this has been a relatively frequent reason for not offering the intestine. This could be overcome by mandatory reporting of the intestinal graft if a donor fits the intestinal donation criteria. Another option could be with a UK-like system where all DBD are potential intestinal donors and are allocated through the system to potential recipients. Thereby reducing the subjectivity of the initial offering process [25]. In general, more awareness needs to be given to the importance of intestinal procurement.

In conclusion, this study makes three points. First, the strict intestinal donor criteria that we had predefined are not routinely followed in our actual practice and -despite that-excellent outcomes are obtained. Second, slightly relaxing intestinal donor criteria and in particular accepting donors with prolonged ICU stay, limited CPR time, and mild inotropic usage can substantially increase the nu mber of offered intestines. Third, the pool of offered intestines is paradoxically underutilized, which is multifactorial in origin. A European intestinal donor organ sharing program should be considered to facilitate donor-recipient matching.

## Data Availability

The original contributions presented in the study are included in the article/Supplementary Material, further inquiries can be directed to the corresponding author.

## Supplementary Material

Supplementary Figure S1 Donor pool (01 January 2014–31 December 2019) filtered by predefined criteria for potential DCD intestinal donors (BMI, Body Mass Index; CPR, Cardiopulmonary Resuscitation; DBD, Donation after Brain Death; ICU, Intensive Care Unit).

Supplementary Figure S2 Donor pool (01 January 2014–31 December 2019) filtered according to effective criteria for potential DCD intestinal donors (BMI, Body Mass Index; CPR, Cardiopulmonary Resuscitation; DBD, Donation after Brain Death; ICU, Intensive Care Unit).

## Abbreviations

[Na+], Serum Sodium Concentration; γ, µg/kg/min; ALT, Alanine Transaminase; AST, Aspartate Transaminase; BMI, Body Mass Index; CMV, Cytomegalovirus; CPR, Cardiopulmonary Resuscitation; DBD, Donation after Brain Death; DCD, Donation after Circulatory Death; ELIAC, ET Liver Intestine Advisory Committee; ET, Eurotransplant; ICU, Intensive Care Unit; INR, International Normalized Ratio; ITx, Intestinal Transplantation; LSGO, Leuven Samenwerkende Groep voor Orgaandonatie (Leuven cooperating group for organ donation); NHS, National Health Service; NRP, Normothermic Regional Perfusion; UNOS, United Network for Organ Sharing; OPTN, Organ Procurement and Transplantation Network.
