# Supplementary material for: Intestinal Donation and Utilization: Single-Center Analysis Within Eurotransplant
Source: Transpl Int. 2023 Aug 21;36:11371. doi: 10.3389/ti.2023.11371 (PMC10476344; doi:10.3389/ti.2023.11371)
Supplement: Supplementary file 1 [file Table1.DOCX]

**Table S1: Overrules of predefined criteria in the actual offered intestines.**

| 33 INTESTINES OFFERED |
| --- |
| PREDEFINED CRITERIA VIOLATIONS (N=20) |
| 10x high inotropic need |
| 5x > 10min CPR |
| 3x > 5 days ICU |
| 1x > 20min hypotension |
| 1x 4 units packed cells |
| OTHER REASONS (N=4) |
| 2x infectious |
| 1x malignancy |
| 1x benign meningioma |

(CPR: Cardiopulmonary Resuscitation; ICU: Intensive Care Unit; N: Number)

**Table S2: Overrules of predefined criteria in the actual transplanted intestines.**

| 10 INTESTINES TRANSPLANTED |
| --- |
| PREDEFINED CRITERIA VIOLATIONS (N=5) |
| 2x high inotropic need |
| 2x > 5 days ICU |
| 1x > 10min CPR |
| OTHER REASONS (N=1) |
| 1x infectious (bacterial meningitis) |

(CPR: Cardiopulmonary Resuscitation; ICU: Intensive Care Unit; N: Number)

**Table S3: Different organizational intestinal organ donor criteria.**

| **INTESTINAL DONOR CRITERIA** | | | | | | | | | | | | | | | | | | | | | |
| --- | --- | --- | --- | --- | --- | --- | --- | --- | --- | --- | --- | --- | --- | --- | --- | --- | --- | --- | --- | --- | --- |
| ORGANIZATION / Author | COUNTRY | YEAR | DONOR TYPE | AGE (years) | Weight | BMI | Blood group compatibility | Size match | Smoking | Alcohol | Drug | Diabetes mellitus | Liver function | Kidney function | Last serum [Na+] | Resuscitation | Inotropes | ICU stay | Cold ischemia time | History of gastro-intestinal disease | Extras |
| ET / ELIAC [12] | Europe | 2014 |  | ≤50 |  | ≤25 kg/m² |  |  |  |  |  |  |  |  |  |  |  | ≤ 7 days |  |  | Lab results equal for liver Tx |
| OPTN/UNOS [13,14] | USA | 2016 | DBD | <50 |  |  |  |  |  |  |  |  | AST & ALT < 500U/L | Serum creat <2mg/dL (or <1mg/dL if <1y old) | <170 mEq/L | <15 minutes after BD declaration | <2 |  | < 9 hours |  | Other organs retrieved |
| NHS [15] | UK | 2018 | DBD | ≤55 | <80 kg |  |  |  |  |  |  |  |  |  |  |  |  |  |  | No chronic GID or intra-abdominal sepsis |  |
| Fischer-Frölich [16] | Germany | 2012 |  | <50 |  | <28 kg/m² | ABO compatible | compatible (no cut off) |  |  |  |  |  |  | <155 mmol/L | <10 minutes |  | < 1 week |  | No abdominal trauma | Start enteral feeding <24h after ICU admission; no significant need for transfusion |
| University Hospitals of Leuven [8] | Belgium | 2018 | DBD | <50 | <80 kg | <25 kg/m² | ABO compatible |  | No | No abuse | No use | No | Acceptable for transplantation | Acceptable for transplantation | <155 mmol/L | No arrest | Dobutamin <5γ/kg/min.; Noradrenalin <0.1γ/kg/min. | < 5 days |  | No previous abdominal surgery or trauma | No acidosis; also possible heart-lung donor |

(ALT: Alanine Transaminase; AST: Aspartate Transaminase; BMI: Body Mass Index; DBD: Donation after Brain Death; ELIAC: ELIAC: ET Liver Intestine Advisory Committee; ET: Eurotransplant; GID: Gastro-Intestinal Disease; ICU: Intensive Care Unit; [Na+]: Serum Sodium Concentration; NHS: National Health Service; OPTN: Organ Procurement and Transplantation Network; UK: United Kingdom; UNOS: United Network for Organ Sharing; USA: United States of America; “γ”: µg/kg/min.
