# Supplementary material for: Intestinal Donation and Utilization: Single-Center Analysis Within Eurotransplant
Source: Transpl Int. 2023 Aug 21;36:11371. doi: 10.3389/ti.2023.11371 (PMC10476344; doi:10.3389/ti.2023.11371)
Supplement: Supplementary file 2 [file Image2.pdf]

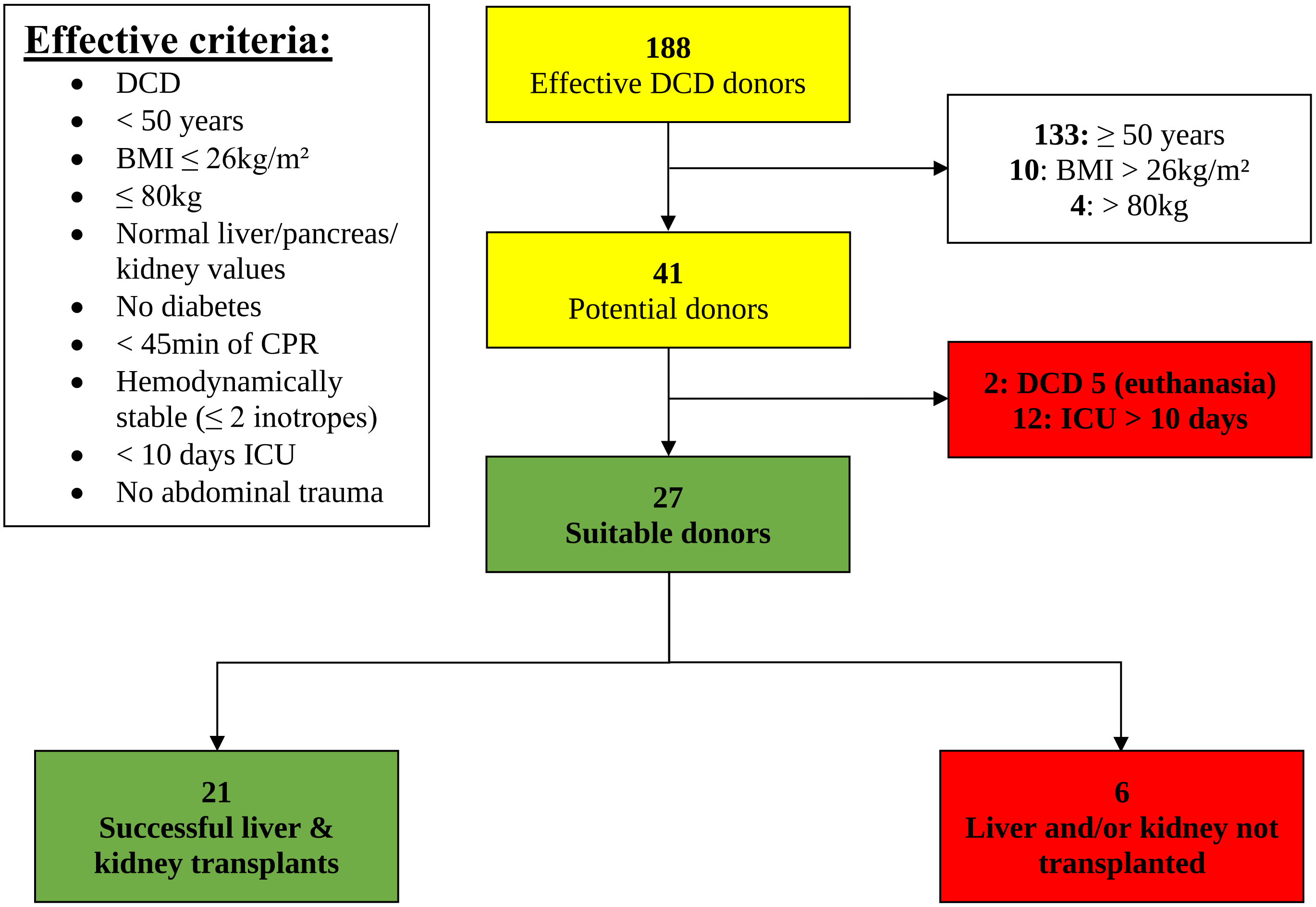
Effective criteria:
DCD
< 50 years
BMI ≤ 26kg/m²
≤ 80kg
Normal liver/pancreas/ kidney values
No diabetes
< 45min of CPR
Hemodynamically stable (≤ 2 inotropes)
< 10 days ICU
No abdominal trauma
188 Effective DCD donors
133: ≥ 50 years
10: BMI > 26kg/m²
4: > 80kg
41 Potential donors
2: DCD 5 (euthanasia)
12: ICU > 10 days
27 Suitable donors
21 Successful liver & kidney transplants
6 Liver and/or kidney not transplanted
