# Supplementary material for: Intestinal Donation and Utilization: Single-Center Analysis Within Eurotransplant
Source: Transpl Int. 2023 Aug 21;36:11371. doi: 10.3389/ti.2023.11371 (PMC10476344; doi:10.3389/ti.2023.11371)
Supplement: Supplementary file 3 [file Image1.pdf]

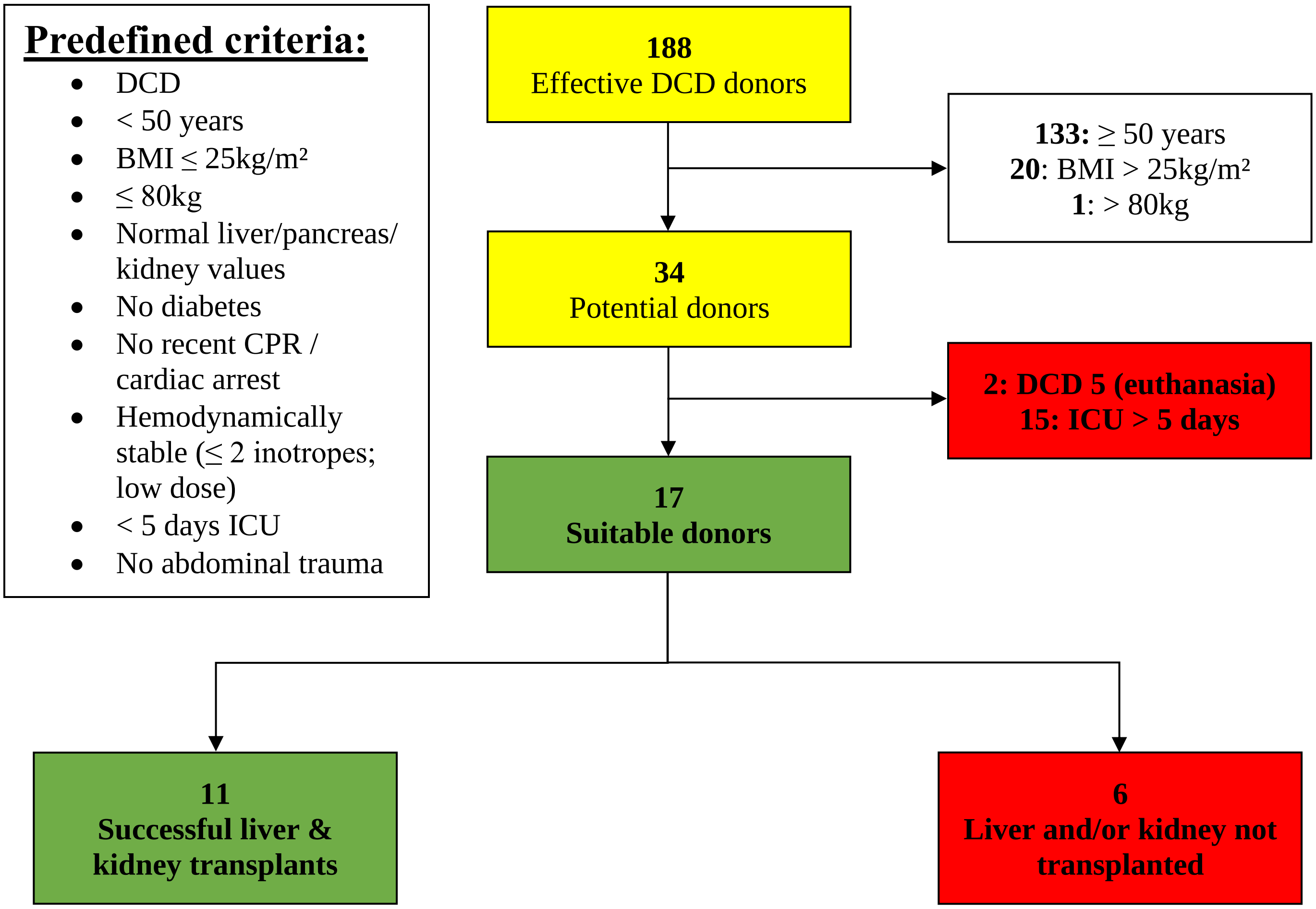
Predefined criteria:
DCD
< 50 years
BMI ≤ 25kg/m²
≤ 80kg
Normal liver/pancreas/ kidney values
No diabetes
No recent CPR / cardiac arrest
Hemodynamically stable (≤ 2 inotropes; low dose)
< 5 days ICU
No abdominal trauma
188 Effective DCD donors
133: ≥ 50 years
20: BMI > 25kg/m²
1: > 80kg
34 Potential donors
2: DCD 5 (euthanasia)
15: ICU > 5 days
17 Suitable donors
11 Successful liver & kidney transplants
6 Liver and/or kidney not transplanted
